# New caddisfly records (Insecta, Trichoptera) for Montenegro and Ecoregion 5, Dinaric Western Balkans: distributional and ecological insights

**DOI:** 10.3897/BDJ.13.e146076

**Published:** 2025-03-25

**Authors:** Hakan Bozdoğan, Astrit Bilalli, Halil Ibrahimi

**Affiliations:** 1 Kırşehir Ahi Evran University, Vocational School of Technical Sciences, Department of Plant and Animal Production, Kırşehir, Turkiye Kırşehir Ahi Evran University, Vocational School of Technical Sciences, Department of Plant and Animal Production Kırşehir Turkiye; 2 Univeristy Haxhi Zeka, Faculty of Agribusiness, Peja, Kosovo Univeristy Haxhi Zeka, Faculty of Agribusiness Peja Kosovo; 3 University of Prishtina, Faculty of Mathematics and Natural Sciences, Department of Biology, Prishtina, Kosovo University of Prishtina, Faculty of Mathematics and Natural Sciences, Department of Biology Prishtina Kosovo; 4 University of Prishtina "Hasan Prishtina", Faculty of Mathematics and Natural Sciences, Department of Biology, Prishtina, Kosovo University of Prishtina "Hasan Prishtina", Faculty of Mathematics and Natural Sciences, Department of Biology Prishtina Kosovo

**Keywords:** caddisflies, aquatic insects, Western Balkans, adult emergence, rare species

## Abstract

**Background:**

The level of knowledge about the caddisfly fauna of Montenegro remains limited compared to that of some other countries in the Balkan Peninsula due to fewer systematic studies and historical gaps in data collection.

**New information:**

Adult caddisfly specimens were collected from the Zeta River in Montenegro during July and October 2024. Additionally, caddisfly data from Montenegro derived from a small, previously unprocessed collection of specimens collected in 2017 were included. Thirteen species belonging to six caddisfly families were identified. Three species are reported for the first time from Montenegro: *Hydroptilaangustata* Mosely, 1939, *Hydropsychebulbifera* McLachlan, 1878 and *Potamophylaxgoulandriourum* Malicky, 1974. All these three species are reported for the first time from Ecoregion 5, Dinaric Western Balkans. Additionally, a few other rarely recorded species in the Western Balkans, such as *Hydropsychemodesta* Navàs, 1925, *Limnephilusgraecus* Schmid, 1965, *Odontocerumalbicorne* (Scopoli, 1763) and *Hydroptilasparsa* Curtis, 1834, were documented. Notably, *Limnephilusgraecus* was found during autumn, despite previously being reported only in spring and summer, suggesting a potential shift in its adult emergence patterns.

These findings enhance our understanding of the distribution and diversity of several rare caddisfly species, particularly with the new records for Montenegro and Ecoregion 5, Dinaric Western Balkans. They underscore the critical need for further research and conservation efforts to support freshwater biodiversity in this region.

## Introduction

Knowledge about the caddisfly fauna of the Western Balkans has increased significantly over the past decade, with numerous new species described; however, less investigated areas still exist. These studies cover a wide array of topics, including taxonomy, molecular analysis, larval description, biogeography and ecological patterns (e.g. Ibrahimi et al. (2012), Oláh and Kovacs (2014), Ibrahimi et al. (2015a), Oláh et al. (2015), Oláh and Beshkov (2016), Ibrahimi et al. (2017), Ibrahimi and Vehapi (2017), Bilalli et al. (2018), Ibrahimi and Sejdiu (2018), Ibrahimi et al. (2019c), Karaouzas et al. (2018), Oláh et al. (2018), Karaouzas et al. (2019), Ibrahimi et al. (2019a), Ibrahimi et al. (2019b), Slavevska-Stamenković et al. (2021), Oláh et al. (2022), Salihu et al. (2023), Bilalli et al. (2024), Ibrahimi et al. (2024b), Musliu et al. (2024)). However, less has been done in caddisfly investigations in Montenegro and the country has been included only sporadically in these studies.

The family Limnephilidae is one of the most ecologically diverse families of caddisflies and most of the recent targeted caddisfly studies in the Balkan Peninsula are focused on this family. Larvae of this family inhabit a wide range of aquatic habitats, including lakes, streams and marshes. Its most diverse genus *Drusus* (Stephens, 1837) has taken increased attention in studies in the Balkan Peninsula, including sporadically Montenegro as well, in recent years (Previšić et al. 2014, Waringer et al. 2015, Ibrahimi et al. 2015b, Ibrahimi et al. 2016b, Oláh et al. 2018). The other genus of this family, *Potamophylax* Wallengren, 1891 has also taken increased attention in studies in the Balkan Peninsula during the few past years with endemic species discovered from all neighbouring countries of Montenegro (Olah and Kovacs 2012, Oláh and Kovács 2013, Oláh and Kovacs 2014, Oláh et al. 2018, Ibrahimi et al. 2021, Ibrahimi et al. 2022, Ibrahimi et al. 2023, Ibrahimi et al. 2024a). Montenegro totally lacks data about diveristy of this genus, while having a huge potential for species of this genus due to the country's diversity of habitats suitable for this genus.

The family Hydroptilidae, commonly known as microcaddisflies, stands out as the most species-rich family within Trichoptera, encompassing over 2,600 described species distributed across six subfamilies and 76 genera, including three fossil genera (Thomson 2023). The genus *Hydroptila* Dalman, 1819 counts for some 5% of the total number of the caddisfly species in the Western Palearctic Region (Ibrahimi 2024). Few targeted studies during the past years from particular areas in the Balkan Peninsula have revealed several low-scale distributed taxa in areas close to Montenegro (Ibrahimi et al. 2014, Ibrahimi et al. 2017, Ibrahimi et al. 2019a, Salihu et al. 2023, Bilalli et al. 2024, Ibrahimi et al. 2024b, Musliu et al. 2024). Despite this, the family remains largely unknown in the Balkan Peninsula in terms of diversity and distribution, including Montenegro. The limited data available on Hydroptilidae likely reflect both the challenging nature of studying these microcaddisflies and the region's historically underexplored biodiversity.

All these mentioned studies in the vicinity of Montenegro show that this country has large potential for discovery of new species or other low-scale distributed species of conservation concern. Such studies will not only enhance our understanding of regional distribution of caddisflies in Montenegro, but also contribute to broader efforts in freshwater biodiversity conservation.

To address the gaps in caddisfly knowledge in Montenegro, we conducted an inventory of caddisfly species at one locality along the Zeta River during two seasons in 2024 and we present these results in this paper. Additionally, we include caddisfly data from Montenegro derived from a small, previously unprocessed collection of specimens collected in 2017. These data, which include three new records for the country and the entire Ecoregion 5, significantly enhance our understanding of caddisfly distributional patterns in the Balkan Peninsula.

## Materials and methods

### Fieldwork, identification and taxonomic work

Adult caddisflies were sampled using a combination of methods, including an entomological net and hand collection during daylight hours, as well as ultraviolet light traps operated during the night, following the protocol outlined by Malicky (2004). The collected specimens were immediately preserved in 90% ethanol and are currently stored at the Department of Biology, Faculty of Mathematics and Natural Sciences, University of Prishtina "Hasan Prishtina," in Prishtinë, Kosovo. Identification of the adult caddisflies was carried out using the taxonomic key provided by Malicky (2004), ensuring accurate species determination. Genitalia images were captured with an Olympus SC53 camera mounted on an Olympus SZX16 stereomicroscope. The photographs were subsequently refined and processed using Adobe Photoshop CC to enhance clarity and detail.

### Sampling area

The Zeta River is the largest tributary of the Morača River, located in central Montenegro. It originates near the town of Nikšić and flows southeast through the Bjelopavlići Valley and the Danilovgrad Municipality. The river is characterised by a meandering course as it traverses this fertile valley, eventually joining the Morača River a few kilometres north of Podgorica, Montenegro's capital city.

The river basin is predominantly fed by karstic springs, contributing to its steady flow and clear waters. The Zeta River’s hydrology is influenced by the surrounding karst terrain, which plays a critical role in groundwater recharge and discharge patterns in the region. The river's course supports a variety of aquatic and riparian habitats, making it an important system for biodiversity studies (Vugdelic et al. 2021).

The Zeta River and its valley provide a key hydrological corridor, with diverse geomorphological features, including plains, alluvial zones and areas of karstic infiltration. These characteristics make it a suitable area for sampling aquatic organisms and investigating ecological and hydrological processes.

Sampling was conducted near Danilovgrad Town (42.630642°N, 19.03266°E, 46 m a.s.l.) (Fig. 1), at a location where a sidestream discharges into the Zeta River. The sidestream substrate consisted of a heterogeneous mixture of pebbles, rocks and sand, with a distinctly fast water flow that created dynamic microhabitats suitable for various aquatic organisms. In contrast, the main river site exhibited slower water flow, with the substrate predominantly composed of small rocks and pebbles, providing a more stable and less turbulent environment.

The Ibër River originates from six springs on the Hajla Mountain in eastern Montenegro and flows northeast, passing through Ibarac, Rozhajë, Radetina and Bac before entering Serbia. In its upper course, the river traverses the southern part of the Rashka District, flowing through a narrow valley surrounded by steep, forested mountains. It receives water from numerous short mountain streams, but lacks significant tributaries in this section (Bajovic and Baur 2024). The river’s course in Montenegro and southern Serbia is characterised by a relatively steep gradient, contributing to its swift flow and erosive potential. The river passes then through Kosovo and again flows into Serbia.

Sampling was carried out near Rozhajë Town (42.8645951°N, 20.218724°E, 947 m a.s.l.) at a location representing an upstream area of the river. The substrate at this site consisted of pebbles, rocks and sand with considerable fast water flow.

## Taxon treatments

### Hydroptila angustata

Mosely, 1939

4C8D6BCA-8F05-5CD1-8DB6-D978280CCE51

#### Materials

- **Type status:** Other material. **Occurrence:** recordedBy: Hakan Bozdogan, Halil Ibrahimi; individualCount: 1; sex: male; lifeStage: adult; occurrenceID: 7E1DEA49-AB3F-5364-920D-C376557D328E; **Location:** continent: Europe; waterBody: Zeta River; country: Montenegro; municipality: Danilovgrad; verbatimLocality: 11.8 km from Danilovgrad town; verbatimCoordinates: 42.630642°N, 19.03266°E; decimalLatitude: 42.630642; decimalLongitude: 19.03266; **Identification:** identifiedBy: Halil Ibrahimi; **Event:** samplingProtocol: UV light trap; samplingEffort: 1 trap-night; eventDate: 22-07-2024; year: 2024; month: 07; day: 22
- **Type status:** Other material. **Occurrence:** recordedBy: Hakan Bozdogan, Halil Ibrahimi; individualCount: 1; sex: male; lifeStage: adult; occurrenceID: 8D62D079-CF9E-52C6-9787-C31D75A4F973; **Location:** continent: Europe; waterBody: Zeta River; country: Montenegro; municipality: Danilovgrad; verbatimLocality: 11.8 km from Danilovgrad town; verbatimCoordinates: 42.630642°N, 19.03266°E; decimalLatitude: 42.630642; decimalLongitude: 19.03266; **Identification:** identifiedBy: Halil Ibrahimi; **Event:** samplingProtocol: entomological net; samplingEffort: 2 observer-hours; eventDate: 22-07-2024; year: 2024; month: 07; day: 22

#### Diagnosis

The male genitalia (Fig. 2) of the observed specimens correspond well with the drawings by Malicky (2004). Right-angled truncated segment X in lateral view along with the inferior appendages with pointed apices and transversal band make it possible to easily distinguish this species and these specimens from closely-related *Hydroptilasimulans* Mosely, 1920.

#### Distribution

Austria, Bulgaria, China, Cyprus, Czechia, Egypt, Greece, Hungary, Iran, Italy, Kazakhstan, Lebanon, Romania, Russia, Spain, Syria, Turkiye, Ukraine and Uzbekistan (Thomson 2023).

#### Ecology

The adult stage of the species is reported across all seasons (Graf et al. 2008). We found it during spring.

#### Conservation

The species has a status of a Critically Endangered species in Czechia (Komzák and Kroča 2019).

### Hydroptila sparsa

Curtis, 1834

2633CDDF-4E2E-5268-AC34-F7AC5779CD7A

#### Materials

- **Type status:** Other material. **Occurrence:** recordedBy: Halil Ibrahimi; individualCount: 1; sex: male; lifeStage: adult; occurrenceID: 127FC4DA-276E-534C-B5F5-1041060CDAD5; **Taxon:** scientificName: Hydroptilasparsa; **Location:** continent: Europe; country: Montenegro; locality: Zeta River; locationRemarks: 11. 8 km far from Danilovgrad town; decimalLatitude: 42.630642; decimalLongitude: 19.03266; georeferenceProtocol: label; **Identification:** identifiedBy: Halil Ibrahimi; dateIdentified: 2025; **Event:** samplingProtocol: UV light trap; eventDate: 22/7/2024; **Record Level:** language: en; collectionID: University of Prishtina, Department of Biology; collectionCode: Trichoptera; basisOfRecord: PreservedSpecimen

#### Diagnosis

The male genitalia of the observed specimens correspond well with the drawings by Malicky (2004).

#### Distribution

Algeria, Austria, Belarus, Bosnia-Herzegovina, Bulgaria, Croatia, Czechia, Denmark, Great Britain, Estonia, Finland, France, Germany, Greece, Hungary, Iran, Ireland, Israel, Italy, Lebanon, Luxembourg, Montenegro, Netherlands, Poland, Portugal, Republic of Croatia, Serbia, Romania, Russia, Serbia, Slovakia, Slovenia, Spain, Sweden, Switzerland, Turkiye and Ukraine (Ibrahimi et al. 2019a, Thomson 2023).

### Lype reducta

(Hagen, 1868)

E1079A01-0D26-523C-BF67-E354EB5C4671

#### Materials

- **Type status:** Other material. **Occurrence:** recordedBy: Halil Ibrahimi; individualCount: 1; sex: male; lifeStage: adult; occurrenceID: D1456568-AF65-5535-A5D6-F75A0B3589BF; **Taxon:** scientificName: Lypereducta; **Location:** continent: Europe; country: Montenegro; locality: Zeta River; locationRemarks: 11. 8 km far from Danilovgrad town; decimalLatitude: 42.630642; decimalLongitude: 19.03266; georeferenceProtocol: label; **Identification:** identifiedBy: Halil Ibrahimi; dateIdentified: 2025; **Event:** samplingProtocol: UV light trap; eventDate: 22/7/2024; **Record Level:** language: en; collectionID: University of Prishtina, Department of Biology; collectionCode: Trichoptera; basisOfRecord: PreservedSpecimen
- **Type status:** Other material. **Occurrence:** recordedBy: Halil Ibrahimi; individualCount: 4; sex: female; lifeStage: adult; occurrenceID: FF313A3B-BA12-5D2F-9553-C91E1004E16D; **Taxon:** scientificName: Lypereducta; **Location:** continent: Europe; country: Montenegro; locality: Zeta River; locationRemarks: 11. 8 km far from Danilovgrad town; decimalLatitude: 42.630642; decimalLongitude: 19.03266; georeferenceProtocol: label; **Identification:** identifiedBy: Halil Ibrahimi; dateIdentified: 2025; **Event:** samplingProtocol: UV light trap; eventDate: 22/7/2024; **Record Level:** language: en; collectionID: University of Prishtina, Department of Biology; collectionCode: Trichoptera; basisOfRecord: PreservedSpecimen

#### Diagnosis

The male genitalia of the observed specimens correspond well with the drawings by Malicky (2004).

#### Distribution

The species has wide distribution across all of Europe and Turkiye as well (Neu et al. 2018, Cerjanec et al. 2020).

### Silo piceus

(Brauer, 1857)

2E847225-E0A7-50FC-9F82-F8D3E919D176

#### Materials

- **Type status:** Other material. **Occurrence:** recordedBy: Halil Ibrahimi; individualCount: 1; sex: male; lifeStage: adult; occurrenceID: E1EB99C5-F7BD-58CC-983E-2E8890A3FBA8; **Taxon:** scientificName: Silopiceus; **Location:** continent: Europe; country: Montenegro; locality: Zeta River; locationRemarks: 11. 8 km far from Danilovgrad town; decimalLatitude: 42.630642; decimalLongitude: 19.03266; georeferenceProtocol: label; **Identification:** identifiedBy: Halil Ibrahimi; dateIdentified: 2025; **Event:** samplingProtocol: UV light trap; eventDate: 22/7/2024; **Record Level:** language: en; collectionID: University of Prishtina, Department of Biology; collectionCode: Trichoptera; basisOfRecord: PreservedSpecimen
- **Type status:** Other material. **Occurrence:** recordedBy: Halil Ibrahimi; individualCount: 2; sex: female; lifeStage: adult; occurrenceID: 9640A77E-867C-5432-B94F-27B3FC4B279B; **Taxon:** scientificName: Silopiceus; **Location:** continent: Europe; country: Montenegro; locality: Zeta River; locationRemarks: 11. 8 km far from Danilovgrad town; decimalLatitude: 42.630642; decimalLongitude: 19.03266; georeferenceProtocol: label; **Identification:** identifiedBy: Halil Ibrahimi; dateIdentified: 2025; **Event:** samplingProtocol: UV light trap; eventDate: 22/7/2024; **Record Level:** language: en; collectionID: University of Prishtina, Department of Biology; collectionCode: Trichoptera; basisOfRecord: PreservedSpecimen

#### Diagnosis

The male genitalia of the observed specimens correspond well with the drawings by Malicky (2004).

#### Distribution

Widely distributed species in Europe (Neu et al. 2018).

### Hydropsyche bulbifera

McLachlan, 1878

9CBBF662-ED63-58DD-96F1-EE32D7FBB1CB

#### Materials

- **Type status:** Other material. **Occurrence:** recordedBy: Halil Ibrahimi; individualCount: 3; sex: male; lifeStage: adult; occurrenceID: AA6E0F8C-7FA8-5843-8805-D745C46B8D32; **Taxon:** scientificName: Hydropsychebulbifera; **Location:** continent: Europe; country: Montenegro; locality: Ibër River; locationRemarks: Upstream segment of Ibër River, near Rozhajë town.; decimalLatitude: 42.864595126785; decimalLongitude: 20.218724075909; georeferenceProtocol: label; **Identification:** identifiedBy: Halil Ibrahimi; dateIdentified: 2025; **Event:** samplingProtocol: sweeping; eventDate: 12/9/2017; **Record Level:** language: en; collectionID: University of Prishtina, Department of Biology; collectionCode: Trichoptera; basisOfRecord: PreservedSpecimen

#### Diagnosis

The male genitalia (Fig. 3) of the observed specimens correspond well with the drawings by Malicky (2004).

#### Distribution

The species is distributed from most of Europe, Turkiye and Iran, but with only few records from south-eastern Europe (Neu et al. 2018).

### Hydropsyche modesta

Navás, 1925

5265B7A8-DA53-5AAA-9C72-48EA5DEBAA5A

#### Materials

- **Type status:** Other material. **Occurrence:** recordedBy: Halil Ibrahimi; individualCount: 1; sex: male; lifeStage: adult; occurrenceID: 35E2D685-763E-5D2D-9C14-033DE674BAD5; **Taxon:** scientificName: Hydropsychemodesta; **Location:** continent: Europe; country: Montenegro; locality: Ibër River; locationRemarks: Upstream segment of Ibër River, near Rozhajë town.; decimalLatitude: 42.864595126785; decimalLongitude: 20.218724075909; georeferenceProtocol: label; **Identification:** identifiedBy: Halil Ibrahimi; dateIdentified: 2025; **Event:** samplingProtocol: sweeping; eventDate: 12/9/2017; **Record Level:** language: en; collectionID: University of Prishtina, Department of Biology; collectionCode: Trichoptera; basisOfRecord: PreservedSpecimen

#### Diagnosis

The male genitalia of the observed specimens correspond well with the drawings by Malicky (2004).

#### Distribution

The species is reported from most of Europe, Turkiye and Syria, with only few records from south-eastern Europe (Neu et al. 2018).

### Grammotaulius nigropunctatus

(Retzius, 1783)

E1006920-4560-5FCD-A49E-FEE82E713756

#### Materials

- **Type status:** Other material. **Occurrence:** recordedBy: Halil Ibrahimi; individualCount: 3; sex: male; lifeStage: adult; occurrenceID: 48F62BDD-1637-5E44-9537-2DC0C193B01B; **Taxon:** scientificName: Grammotauliusnigropunctatus (Retzius, 1783); **Location:** continent: Europe; country: Montenegro; locality: Zeta River; locationRemarks: 11. 8 km far from Danilovgrad town; decimalLatitude: 42.630642; decimalLongitude: 19.03266; georeferenceProtocol: label; **Identification:** identifiedBy: Halil Ibrahimi; dateIdentified: 2025; **Event:** samplingProtocol: UV light trap; eventDate: 12/10/2024; **Record Level:** language: en; collectionID: University of Prishtina, Department of Biology; collectionCode: Trichoptera; basisOfRecord: PreservedSpecimen
- **Type status:** Other material. **Occurrence:** recordedBy: Halil Ibrahimi; individualCount: 1; sex: female; lifeStage: adult; occurrenceID: 7856D6A4-0664-50FE-9B52-B3D238A8C42F; **Taxon:** scientificName: Grammotauliusnigropunctatus (Retzius, 1783); **Location:** continent: Europe; country: Montenegro; locality: Zeta River; locationRemarks: 11. 8 km far from Danilovgrad town; decimalLatitude: 42.630642; decimalLongitude: 19.03266; georeferenceProtocol: label; **Identification:** identifiedBy: Halil Ibrahimi; dateIdentified: 2025; **Event:** samplingProtocol: UV light trap; eventDate: 12/10/2024; **Record Level:** language: en; collectionID: University of Prishtina, Department of Biology; collectionCode: Trichoptera; basisOfRecord: PreservedSpecimen

#### Diagnosis

The male genitalia of the observed specimens correspond well with the drawings by Malicky (2004).

#### Distribution

The species is reported from most of Europe and Turkiye as well (Neu et al. 2018).

### Halesus digitatus

(von Paula Schrank, 1781)

2B91C3E6-FD71-55B5-9BB3-AF3A199EC96E

#### Materials

- **Type status:** Other material. **Occurrence:** recordedBy: Halil Ibrahimi; individualCount: 2; sex: male; lifeStage: adult; occurrenceID: 66D5BF9D-4098-56B1-BBFF-9283E73D49C6; **Taxon:** scientificName: Halesusdigitatus; **Location:** continent: Europe; country: Montenegro; locality: Zeta River; locationRemarks: 11. 8 km far from Danilovgrad town; decimalLatitude: 42.630642; decimalLongitude: 19.03266; georeferenceProtocol: label; **Identification:** identifiedBy: Halil Ibrahimi; dateIdentified: 2025; **Event:** samplingProtocol: UV light trap; eventDate: 12/10/2024; **Record Level:** language: en; collectionID: University of Prishtina, Department of Biology; collectionCode: Trichoptera; basisOfRecord: PreservedSpecimen
- **Type status:** Other material. **Occurrence:** recordedBy: Halil Ibrahimi; individualCount: 3; sex: female; lifeStage: adult; occurrenceID: CDAF9B7D-340A-550C-BB3A-0240349F1770; **Taxon:** scientificName: Halesusdigitatus; **Location:** continent: Europe; country: Montenegro; locality: Zeta River; locationRemarks: 11. 8 km far from Danilovgrad town; decimalLatitude: 42.630642; decimalLongitude: 19.03266; georeferenceProtocol: label; **Identification:** identifiedBy: Halil Ibrahimi; dateIdentified: 2025; **Event:** samplingProtocol: UV light trap; eventDate: 12/10/2024; **Record Level:** language: en; collectionID: University of Prishtina, Department of Biology; collectionCode: Trichoptera; basisOfRecord: PreservedSpecimen

#### Diagnosis

The male genitalia of the observed specimens correspond well with the drawings by Malicky (2004).

#### Distribution

The nominate subspecies is distributed in most of Europe, with two other subspecies known from Caucasus, Iran and Turkiye (Neu et al. 2018).

### Limnephilus graecus

Schmid, 1965

F4C719B8-2C0E-5FE0-8414-DE036AF9E7ED

#### Materials

- **Type status:** Other material. **Occurrence:** sex: 2 males, 1 female; lifeStage: adult; occurrenceID: 38015E14-71E9-52E9-9816-6A325BE4BB11; **Location:** continent: Europe; waterBody: Zeta River; country: Montenegro; municipality: Danilovgrad; locality: 11. 8 km far from Danilovgrad town; verbatimLocality: 42.630642°N, 19.03266°E; **Event:** samplingProtocol: UV light trap; eventDate: 22-10-2024

#### Diagnosis

The male (Fig. 4) and female genitalia of the observed specimens correspond with drawings of Malicky (2004).

#### Distribution

Greece, Albania, Montenegro, Croatia (Neu et al. 2018).

#### Ecology

The adult stage of the species is reported in spring and summer (Graf et al. 2008) and we found it during October.

### Micropterna sequax

McLachlan, 1875

6435EE39-9DB6-5C44-BE7F-BDC4B13C9B8B

#### Materials

- **Type status:** Other material. **Occurrence:** recordedBy: Halil Ibrahimi; individualCount: 4; sex: male; lifeStage: adult; occurrenceID: C9B622B2-5CEF-598F-9350-FE0261844887; **Taxon:** scientificName: Micropternasequax McLachlan, 1875; **Location:** continent: Europe; country: Montenegro; locality: Zeta River; locationRemarks: 11. 8 km far from Danilovgrad town; decimalLatitude: 42.630642; decimalLongitude: 19.03266; georeferenceProtocol: label; **Identification:** identifiedBy: Halil Ibrahimi; dateIdentified: 2025; **Event:** samplingProtocol: UV light trap; eventDate: 12/10/2024; **Record Level:** language: en; collectionID: University of Prishtina, Department of Biology; collectionCode: Trichoptera; basisOfRecord: PreservedSpecimen
- **Type status:** Other material. **Occurrence:** recordedBy: Halil Ibrahimi; individualCount: 7; sex: female; lifeStage: adult; occurrenceID: 66A4FDC0-EB20-58E4-A7DD-6EF336CEA6FE; **Taxon:** scientificName: Micropternasequax McLachlan, 1875; **Location:** continent: Europe; country: Montenegro; locality: Zeta River; locationRemarks: 11. 8 km far from Danilovgrad town; decimalLatitude: 42.630642; decimalLongitude: 19.03266; georeferenceProtocol: label; **Identification:** identifiedBy: Halil Ibrahimi; dateIdentified: 2025; **Event:** samplingProtocol: UV light trap; eventDate: 12/10/2024; **Record Level:** language: en; collectionID: University of Prishtina, Department of Biology; collectionCode: Trichoptera; basisOfRecord: PreservedSpecimen

#### Diagnosis

The male genitalia of the observed specimens correspond well with the drawings by Malicky (2004).

#### Distribution

Widely distributed species in most of Europe and Turkiye as well (Neu et al. 2018).

### Potamophylax goulandriorum

Malicky, 1975 sp. nov.

2804BD5F-2D7C-5D92-A943-3D8D9680214D

#### Diagnosis

**Figure 5.**
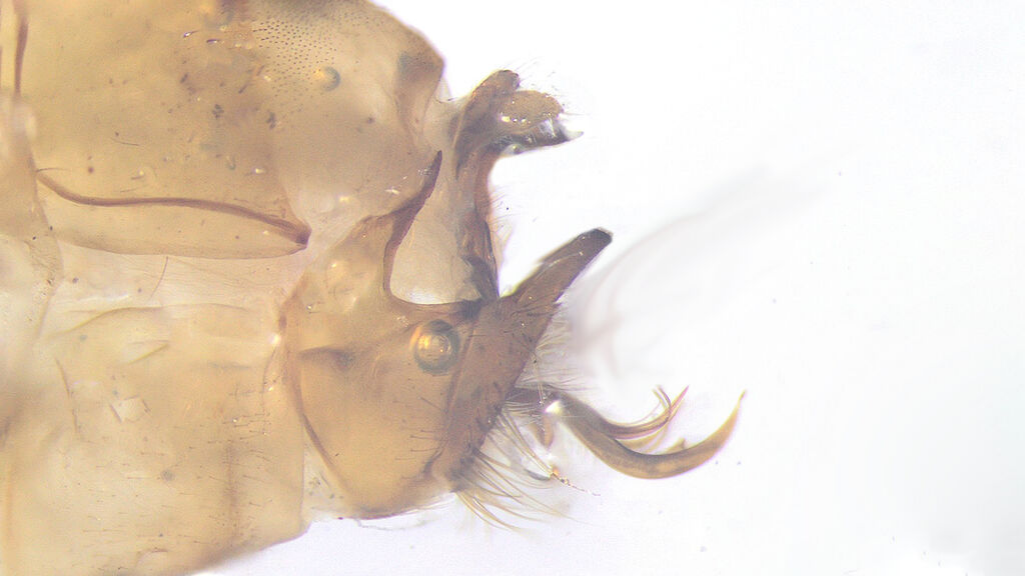
Lateral view of male genitalia of *Potamophylaxgoulandriorum*.

The male genitalia (Fig. 5) of the observed specimens correspond well with the drawings by Malicky (2004).

#### Distribution

A Balkan endemic species (Neu et al. 2018).

### Potamophylax pallidus

(Klapalek, 1899)

ADE07FA8-E037-5674-898C-32DCD3C03093

#### Materials

- **Type status:** Other material. **Occurrence:** recordedBy: Halil Ibrahimi; individualCount: 1; sex: male; lifeStage: adult; occurrenceID: 328475AF-2735-5021-8182-337E757D5497; **Taxon:** scientificName: Potamophylaxpallidus (Klapalek, 1899); **Location:** continent: Europe; country: Montenegro; locality: Zeta River; locationRemarks: 11. 8 km far from Danilovgrad town; decimalLatitude: 42.630642; decimalLongitude: 19.03266; georeferenceProtocol: label; **Identification:** identifiedBy: Halil Ibrahimi; dateIdentified: 2025; **Event:** samplingProtocol: UV light trap; eventDate: 12/10/2024; **Record Level:** language: en; collectionID: University of Prishtina, Department of Biology; collectionCode: Trichoptera; basisOfRecord: PreservedSpecimen
- **Type status:** Other material. **Occurrence:** recordedBy: Halil Ibrahimi; individualCount: 4; sex: female; lifeStage: adult; occurrenceID: 25E60E71-714F-54ED-8F6C-0E8D197A4FC3; **Taxon:** scientificName: Potamophylaxpallidus (Klapalek, 1899); **Location:** continent: Europe; country: Montenegro; locality: Zeta River; locationRemarks: 11. 8 km far from Danilovgrad town; decimalLatitude: 42.630642; decimalLongitude: 19.03266; georeferenceProtocol: label; **Identification:** identifiedBy: Halil Ibrahimi; dateIdentified: 2025; **Event:** samplingProtocol: UV light trap; eventDate: 12/10/2024; **Record Level:** language: en; collectionID: University of Prishtina, Department of Biology; collectionCode: Trichoptera; basisOfRecord: PreservedSpecimen

#### Diagnosis

The male genitalia of the observed specimens correspond well with the drawings by Malicky (2004).

#### Distribution

The species has a limmited distribution mostly confined to south-eastern Europe, Central Europe with a single report from Turkiye (Neu et al. 2018).

### Odontocerum albicorne

(Scopoli, 1763)

BF1F6A2F-F9B9-5DC3-B92D-062CF399DA91

#### Materials

- **Type status:** Other material. **Occurrence:** recordedBy: Halil Ibrahimi; individualCount: 5; sex: male; lifeStage: adult; occurrenceID: 90FC9CBF-E6E4-5F92-9120-A37F02607989; **Taxon:** scientificName: Odontocerumalbicorne; **Location:** continent: Europe; country: Montenegro; locality: Zeta River; locationRemarks: 11. 8 km far from Danilovgrad town; decimalLatitude: 42.630642; decimalLongitude: 19.03266; georeferenceProtocol: label; **Identification:** identifiedBy: Halil Ibrahimi; dateIdentified: 2025; **Event:** samplingProtocol: UV light trap; eventDate: 22/7/2024; **Record Level:** language: en; collectionID: University of Prishtina, Department of Biology; collectionCode: Trichoptera; basisOfRecord: PreservedSpecimen
- **Type status:** Other material. **Occurrence:** recordedBy: Halil Ibrahimi; individualCount: 9; sex: female; lifeStage: adult; occurrenceID: 0E072DA0-4C8E-5B3D-8047-FD4A72E5D036; **Taxon:** scientificName: Odontocerumalbicorne; **Location:** continent: Europe; country: Montenegro; locality: Zeta River; locationRemarks: 11. 8 km far from Danilovgrad town; decimalLatitude: 42.630642; decimalLongitude: 19.03266; georeferenceProtocol: label; **Identification:** identifiedBy: Halil Ibrahimi; dateIdentified: 2025; **Event:** samplingProtocol: UV light trap; eventDate: 22/7/2024; **Record Level:** language: en; collectionID: University of Prishtina, Department of Biology; collectionCode: Trichoptera; basisOfRecord: PreservedSpecimen

#### Diagnosis

The male genitalia of the observed specimens correspond well with the drawings by Malicky (2004).

#### Distribution

Widely distributed species in Europe, but with only few records from south-eastern Europe (Neu et al. 2018).

## Discussion

Despite extensive caddisfly studies from neighbouring countries, such as Albania and Kosovo, only few studies emerged recently from Montenegro (Oláh and Kovács 2013, Oláh and Kovacs 2014, Karaouzas et al. 2019, Ibrahimi et al. 2019a). Currently, only 144 species are known from Montenegro (Karaouzas et al. 2019, Ibrahimi et al. 2022) which is a very low number considering the size of the country and diversity of freshwater habitats. The country has potential for many more species as documented by this study, limited in time and number of sampling sites, where we found three first country records, all of them reported for the first time from Ecoregion 5, Dinaric Western Balkans.

*Hydroptilaangustata* was originally described from Egypt and has since been documented across a relatively wide range in the Western Palearctic Region. Within the Balkan Peninsula, it has been reported from Bulgaria, Greece and Albania, yet remains unrecorded in most neighbouring countries of Montenegro (Thomson 2023). The species was collected exclusively during spring in the Zeta River, despite literature suggesting (Graf et al. 2008) that its adult stage is present throughout all four seasons. Notably, the population density was very low, with only two specimens observed. This lack of information regarding the Hydroptilidae family in Montenegro reflects a general lack of information related to the Trichoptera in general.

*Hydropsychebulbifera* was originally described from Austria and has since been recorded in several regions across the Eastern Mediterranean and Western Palearctic. Within the Balkan Peninsula, it has a limmited distribution (Ibrahimi et al. 2012, Ibrahimi et al. 2014, Ibrahimi et al. 2017, Kučinić et al. 2019). The species was collected during autumn in the Ibër River, a time when its adult emergence aligns with known activity patterns reported in literature (Graf et al. 2008). However, its population density in the surveyed area was exceptionally low, with only three specimens documented. The other species of the Hydropsychidae family found during this investigation, *Hydropsychemodesta*, is reported only for the second time from Montenegro.

*Potamophylaxgoulandriorum* was originally described from Greece and is known for its restricted distribution in the Balkans. While the species has been documented in neighbouring countries such as Albania, Kosovo and North Macedonia (Ibrahimi et al. 2016a), it has not been previously recorded in Montenegro. Specimens were collected during autumn along the Ibër River, consistent with its typical adult flight period. The population density was notably low, with only a single specimen observed. This discovery highlights the limited knowledge of the *Potamophylax* genus in Montenegro and points to broader gaps in understanding of Trichoptera biodiversity in the region.

*Limnephilusgraecus*, a rare and endemic species of the Balkan Peninsula, is infrequently reported. Literature data indicate that the adult stage of this species typically occurs in spring and summer. Interestingly, we documented *L.graecus* during autumn, raising questions about its seasonal activity. While this observation might reflect a lack of targeted studies during autumn, it is also possible that climate change is influencing the flight period of the species. It is worth noting that the collection period during autumn coincided with atypical spring-like weather conditions and a similar pattern has been noted for quite some years in many parts of the Balkan Peninsula. In aquatic ecosystems, rising water temperatures can impact invertebrate survival and alter the timing of critical events like egg hatching. A recent study (Shackleton et al. 2024) suggested that relevant changes in water and air temperature, will most certainly impact the emergence period of aquatic insects and, consequently, impact a whole chain of prey-predator interactions considering the fact that aquatic insects through their adult emergence patterns play a significant role in energy transfer between aquatic and terrestrial ecosystems.

This study also contributes to the range extension of several other species found during this investigation, such as: *Hydroptilasparsa* and *Odontocerumalbicorne. Hydroptilasparsa* is relatively widespread in Europe, but mostly in Central and Western Europe and Greece (Neu et al. 2018). In Western Balkan countries, there are relatively only few records of this species. As stated above for *Hydroptilaangustata*, the reason for this may be the low level of investigations in habitats suitable for Hydroptilids in this area. Our finding of *Hydroptilasparsa* represents only the second record of this species in Montenegro, the first one being in Buna River in Shtodër Village (Ibrahimi et al. 2019a). In the Balkans, it is reported also from Albania, Bosnia and Herzegovina, Serbia and much of Greece (Thomson 2023), while in Kosovo, it is reported only as Hydroptilagr.sparsa, based on a single female specimen (Ibrahimi et al. 2014).

*Odontocerumalbicorne*, a rather widespread species in much of Europe is reported, however, only from few localities in the Western Balkans. Although reported from all Balkan countries, this study contributes to the knowledge of the range expansion of this species in Montenegro. The finding of *Lypereducta*, a relatively widespread species in Europe (Ibrahimi et al. 2014, Neu et al. 2018), also contributes to the range extension of this species in Montenegro.

This investigation, despite being based on a limited sampling effort, yielded significant insights into the biogeography and ecology of caddisflies in Montenegro. The findings underscore the country's potential as a habitat for rare and ecologically significant caddisfly species. These results highlight the critical need for more comprehensive studies in Montenegro to better understand and conserve its unique aquatic biodiversity.

## Supplementary Material

XML Treatment for Hydroptila angustata

XML Treatment for Hydroptila sparsa

XML Treatment for Lype reducta

XML Treatment for Silo piceus

XML Treatment for Hydropsyche bulbifera

XML Treatment for Hydropsyche modesta

XML Treatment for Grammotaulius nigropunctatus

XML Treatment for Halesus digitatus

XML Treatment for Limnephilus graecus

XML Treatment for Micropterna sequax

XML Treatment for Potamophylax goulandriorum

XML Treatment for Potamophylax pallidus

XML Treatment for Odontocerum albicorne

## Figures and Tables

**Figure 1a.**
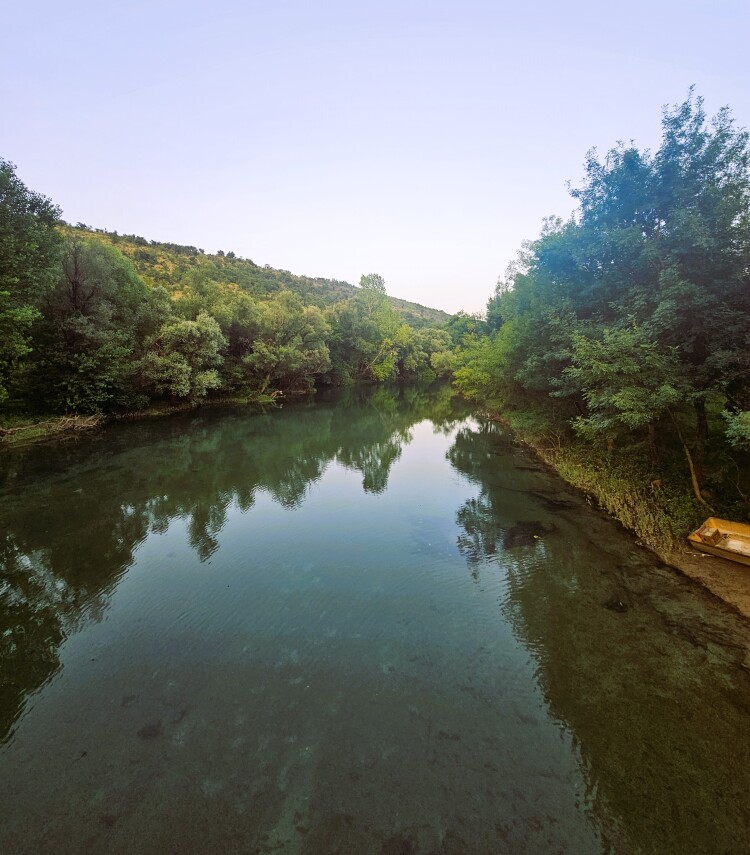
Overview of the study site;

**Figure 1b.**
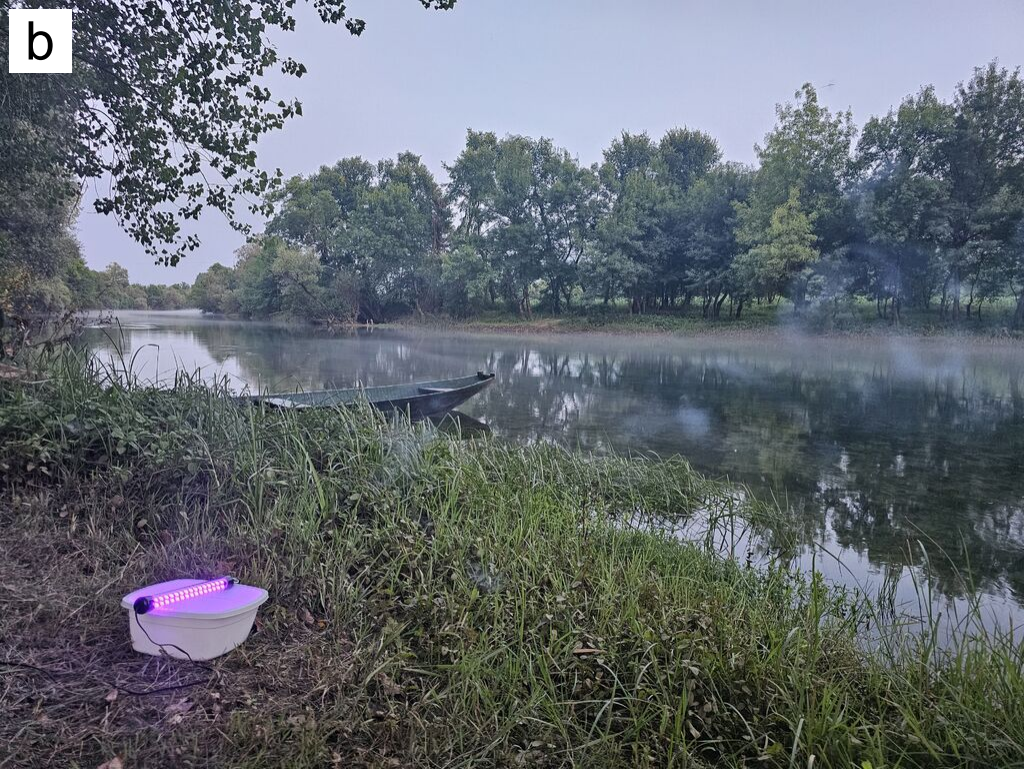
Operation of UV-light trap at the study site.

**Figure 2a.**
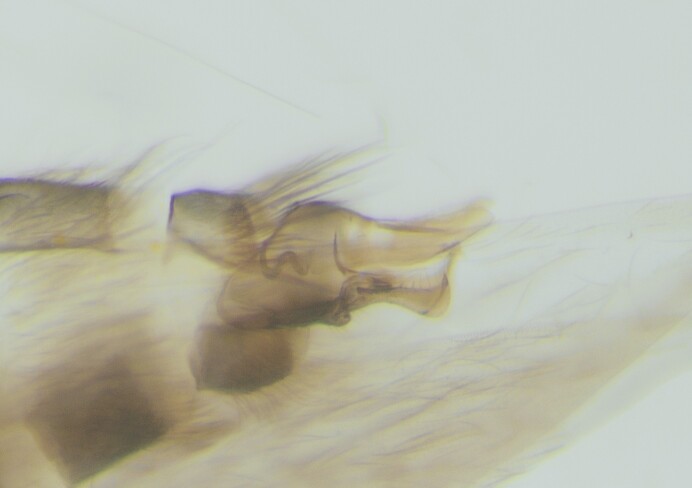
Lateral view;

**Figure 2b.**
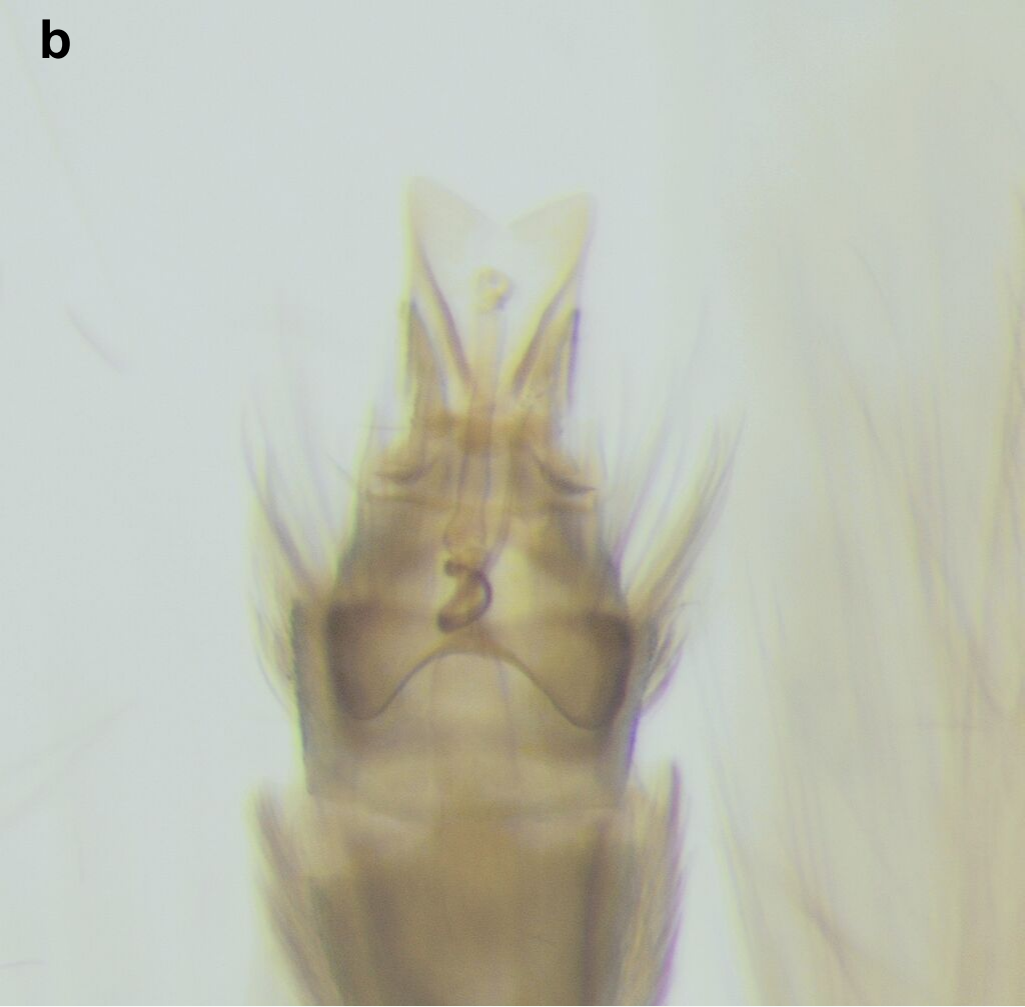
Dorsal view.

**Figure 3a.**
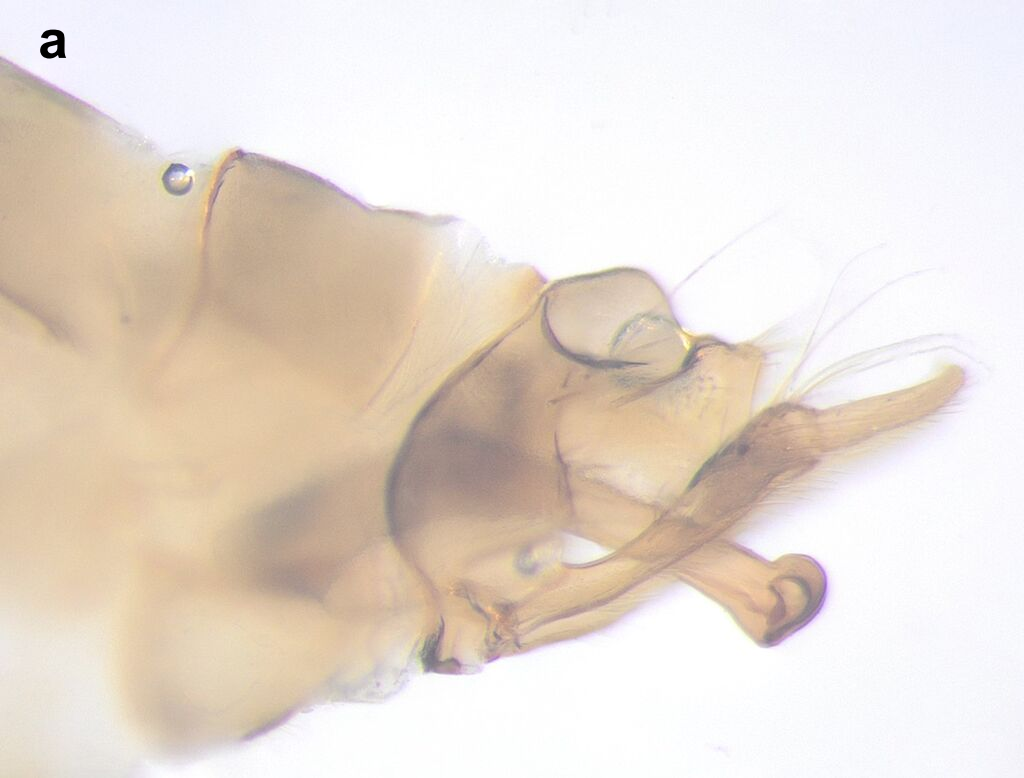
Lateral view;

**Figure 3b.**
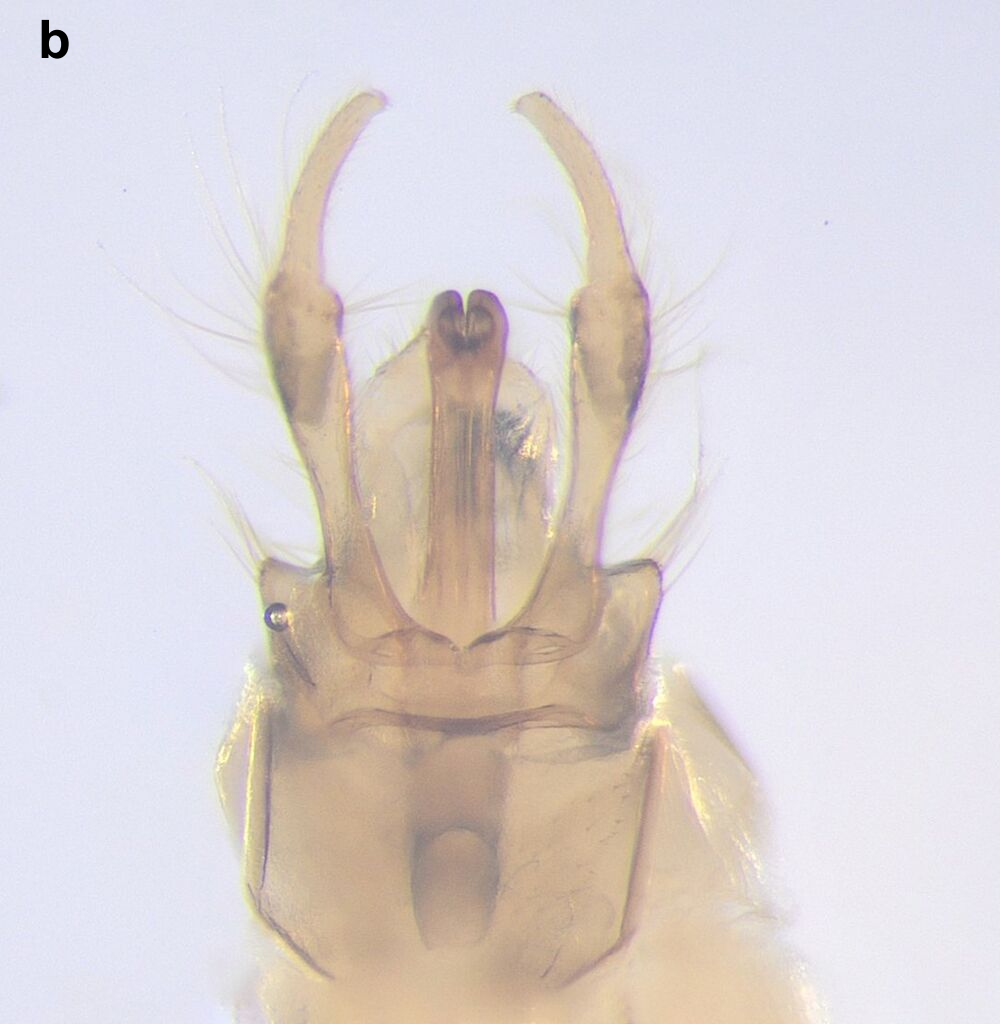
Ventral view.

**Figure 4a.**
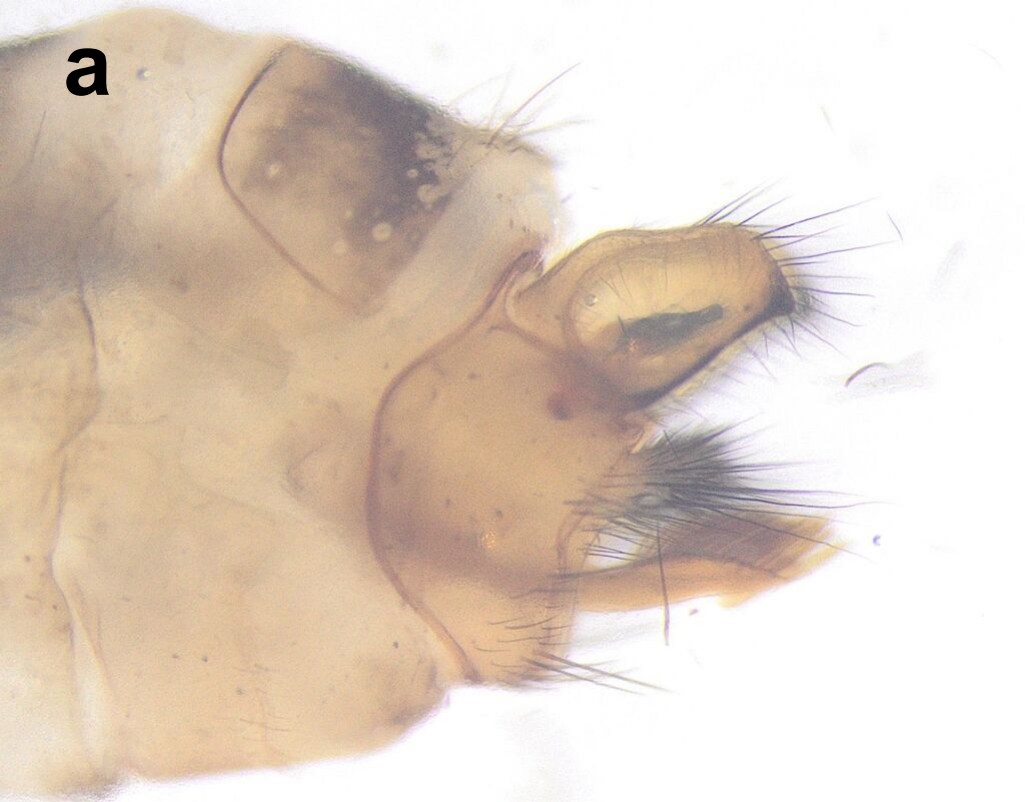
Lateral view;

**Figure 4b.**
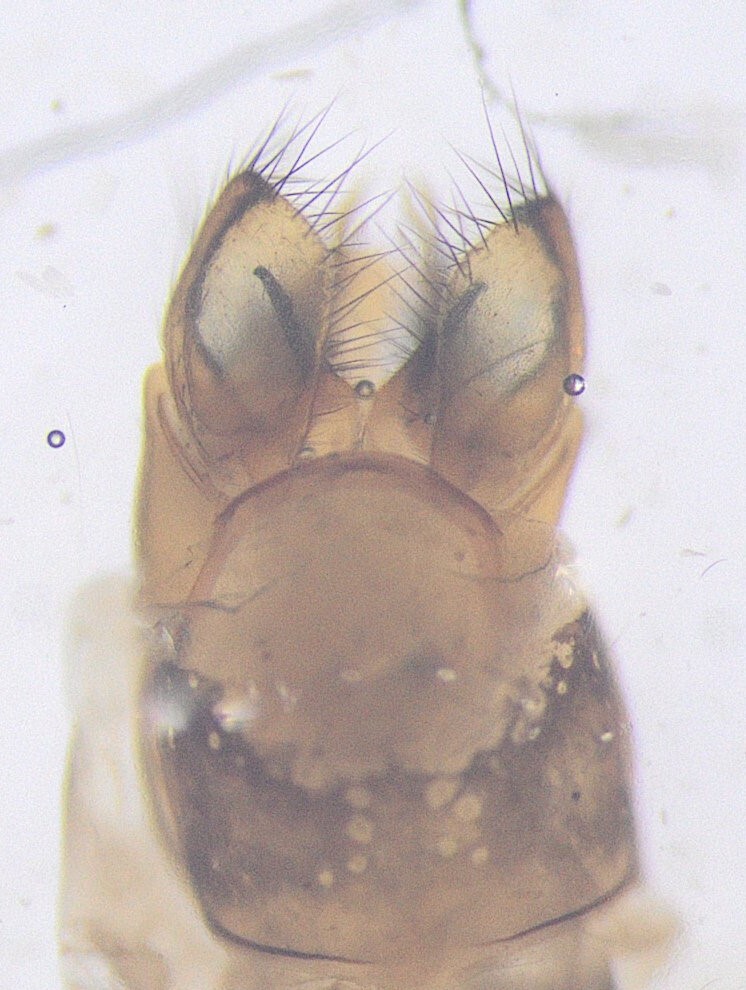
Dorsal view;

**Figure 4c.**
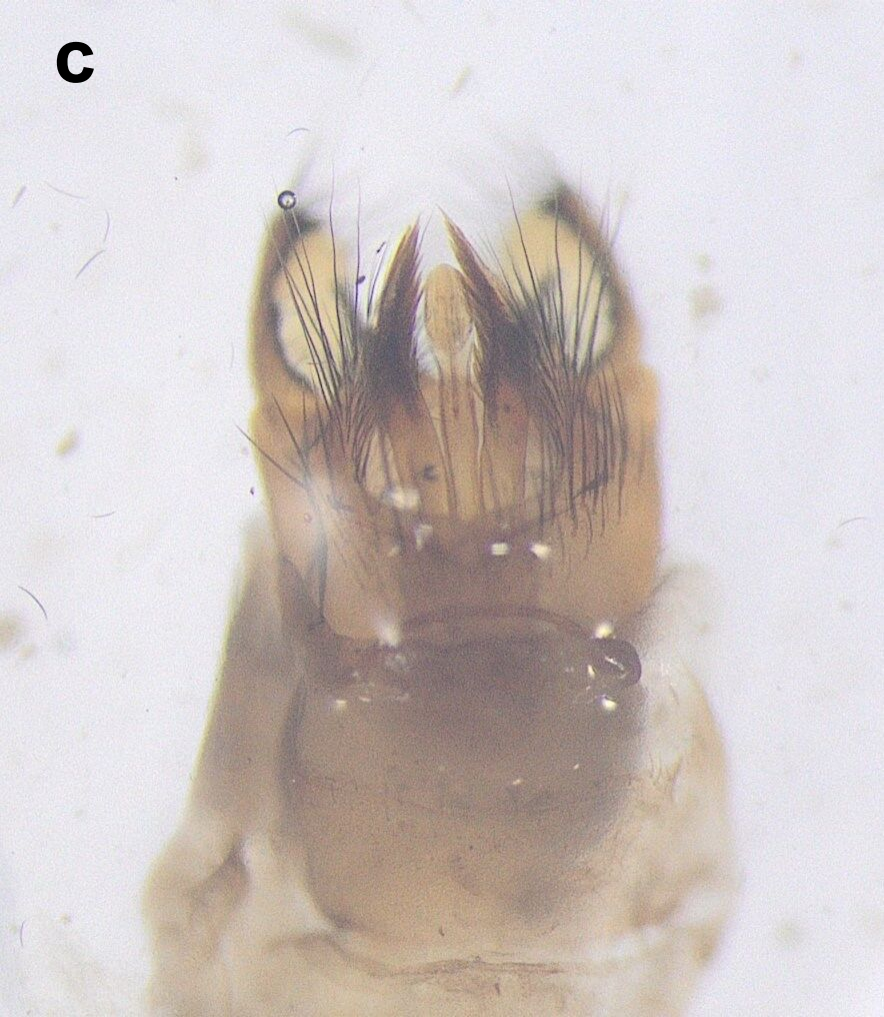
Ventral view.
